# Review of the genus *Liocrobyla* (Lepidoptera, Gracillariidae, Ornixolinae) from Korea, with description of one newly-recorded species

**DOI:** 10.3897/BDJ.11.e115509

**Published:** 2023-12-08

**Authors:** Da-Som Kim, Ji-Young Lee, Bong-Kyu Byun

**Affiliations:** 1 National Science Museum of Korea, Daejeon, Republic of Korea National Science Museum of Korea Daejeon Republic of Korea; 2 Hannam University, Daejeon, Republic of Korea Hannam University Daejeon Republic of Korea

**Keywords:** Gracillariidae, Ornixolinae, *Liocrobyla*, new record, leafminer, Korea

## Abstract

**Background:**

*Liocrobyla* Meyrick, 1916 is a relatively small genus within the family Gracillariidae, consisting of nine species worldwide, including five species in Korea.

**New information:**

In this study, we recognise five species belonging to the genus *Liocrobyla* Meyrick, 1916 from Korea. Amongst them, one species, *L.indigofera* Liu, Wang & Wang, 2018, is reported for the first time in Korea. Figures of adults, male and female genitalia, along with a key to the species of *Liocrobyla* in Korea, are provided.

## Introduction

The genus *Liocrobyla* was established by Meyrick (1916), with *Liocrobylaparaschista* Meyrick, 1916 as its type species. This genus is a rather small group with only nine described species worldwide (De Prins and De Prins 2011). In Korea, two species: *L.brachybotrys* Kuroko, 1960 and *L.lobota* Kuroko, 1960 were first reported by Park et al. (1983). One species, *L.kumatai* Kuroko, 1982 was added by Park and Han (1986) and one newly-recorded species *L.desmodiella* Kuroko, 1982 was reported by Kim and Byun (2019) for the first time from Korea. In total, only four species of the genus have been recorded from Korea until this study. This study aimed to review and enumerate the Korean species of the genus *Liocrobyla*, including a newly-recorded species. All available information including images of adults, male and female genitalia, host plants and distributional ranges is provided in a catalogue of Korea.

## Materials and methods

The specimens examined in this study were deposited in the Systematic Entomology Laboratory, Hannam University, Daejeon, Korea (HNSUEL). Male and female genitalia were dissected and mounted with Euparal mountant, following the procedure described in Holloway et al. (1987). Images of the adult were taken using a digital camera (Canon EOS 600D, Canon Inc., Ota, Tokyo, Japan) and images of genitalia were captured using a digital camera attached to the microscope, LEICA M205C, Leica Microsystems, Wetzlar, Hesse, Germany) and refined in Photoshop CS5 software.

Abbreviations in this study for the locality in Korea are as follows: TL (type locality), TD (type depository). Additionally, the specimen depositories in this study were examined from the following collections: HNUSEL, Systematic Entomology Laboratory, Hannam University, Korea; INU Department of Life Sciences, Incheon National University, Korea; KNAE Korea National Insect Collection, Korea National Arboretum, Korea; NAIST/NAAS National Academy of Agricultural Science, Korea; EIHU Entomological Institute, Hokkaido University, Japan; ELKU Entomological Laboratory Kyushu University, Japan; NHMUK The Natural History Museum, London, United Kingdom; SDNU Zoological Collection, Shandong Normal University, China.

## Taxon treatments

### Liocrobyla brachybotrys

Kuroko, 1960

7FDA3ADB-2EA4-5D41-837C-E6AB61B6D11A

Liocrobyla brachybotrys Kuroko, 1960: 6. TL: Kyushu, Japan. TD: ELKU (Holotype; Allotype); ELKU, NHMUK (Paratypes).

#### Materials

- **Type status:** Other material. **Occurrence:** individualCount: 1; sex: male; otherCatalogNumbers: gen.slide no. HNUSEL-5529; **Taxon:** taxonID: HNU_GRD_0195; scientificNameID: *Liocrobylabrachybotrys* Kuroko, 1960; phylum: Arthropoda; class: Insecta; order: Lepidoptera; family: Gracillariidae; genus: Liocrobyla ; **Location:** country: South Korea; stateProvince: Gyeonsangbuk-do; county: Gyeongju-si; **Event:** year: 1980; month: 8; day: 22

#### Description

##### Redescription

**Figure 1.**
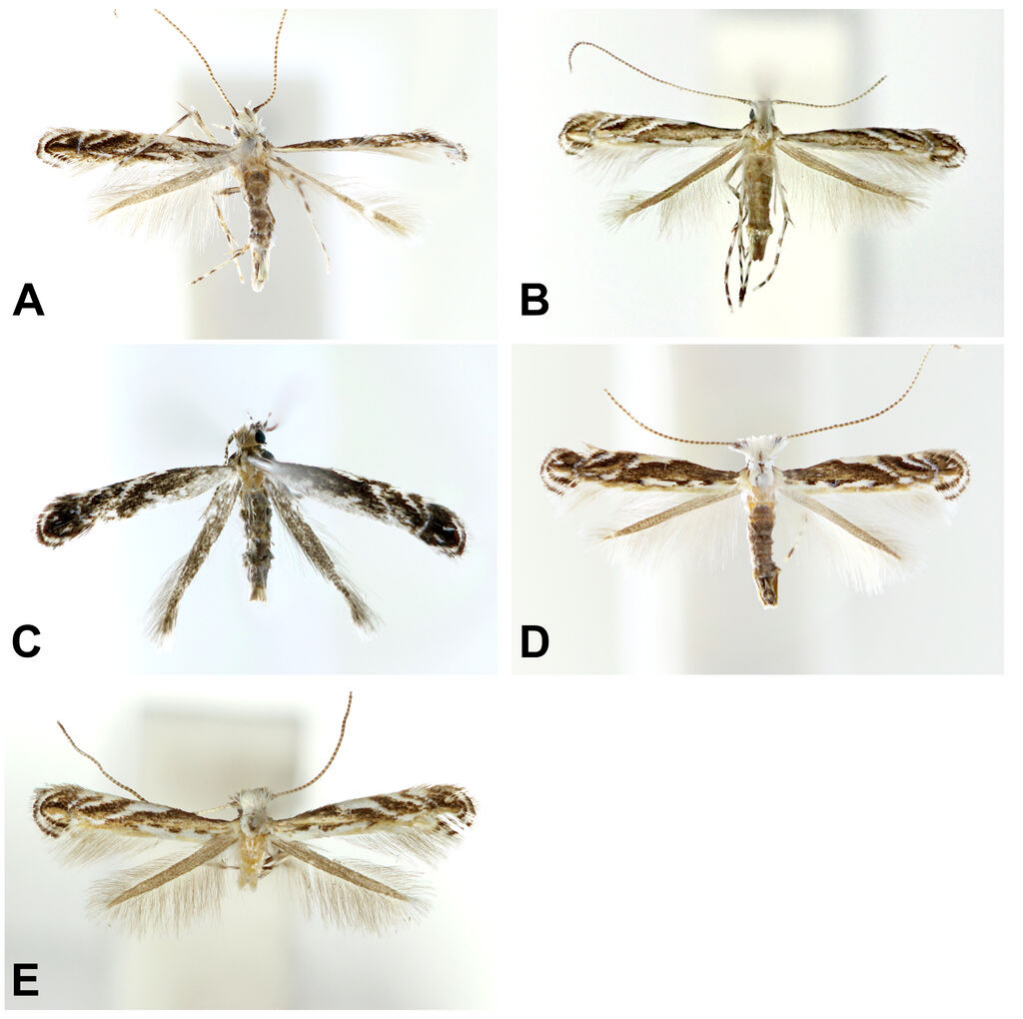
Adults of genus *Liocrobyla*. **A.** *L.brachybotrys* (gen. slide no. HNUSEL-5529); **B.** *L.desmodiella* (gen. slide no. HNUSEL-5321); **C.** *L.indigofera* (gen. slide no. HNUSEL-5530); **D.** *L.kumatai* (gen. slide no. HNUSEL-5528); **E.** *L.lobata* (gen. slide no. HNUSEL-5320).

**Adult** (Fig. 1A). Head a tuft of fuscous scales near scape; frons white and pale ochrous with a tuft of white rather long scales below scape and the scales basal fuscous; maxillary palpus white with fuscous laterally; labial palpus white and apical of second segment with a fuscous band; antenna fuscous dorsally and white ventrally; scape fuscous dorsally and white on ventrally with a tuft scales on below. Thorax white mixed with pale ochrous; legs white with rough scales; fore coxa greyish-brown; middle femur and tibia fuscous and two narrow rings on sub-basal and median part of tibia; middle tarsus with four fuscous bands and the last of the two on apex side by side; hind femur greyish-brown; hind tibia white, except for basal and apical part; hind tarsus with three obscure fuscous bands. Wingspan 6.8 mm. Forewing ground colour fuscous mixed with ochrous and blackish scales; a goldish-orange ochrous stripe on the dorsal margin of basal to apical part with some white spots; a short white stria at 1/5 of forewing beginning at the dorsal margin and obliquely stretched outwards; longer white stria at 1/3 of forewing; first costa-stria at half of the forewing, reaching up to wing fold, broadening at basal and narrowing to apical; second costa-stria 2/3 of forewing with a black stria inside; the last white stria minute, curved to the inside and rather longitudinally reaching to wing fold; a pair of white spots on the apex with blackish-brown spot; outer line of apex white with blackish edges on both sides. Hind-wing greyish-brown, cilia long and densely along outer margin.

**Figure 2.**
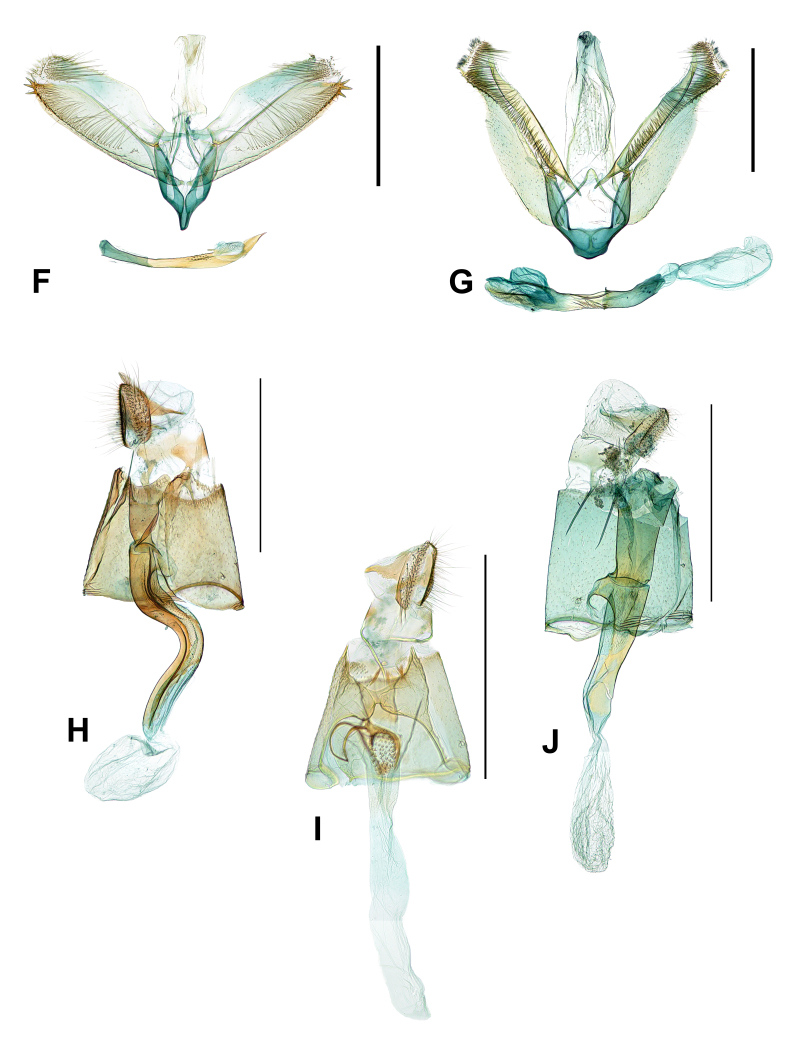
Male and Female genitalia of *Liocrobyla*: **F.** *L.brachybotrys* (♂, gen. slide no. HNUSEL-5529), **G.** *L.desmodiella* (♂, gen. slide no. HNUSEL-5319), **H.** *L.indigofera* (♀, gen. slide no. HNUSEL-5530), **I.** *L.kumatai* (♀, gen. slide no. HNUSEL-5528), **J.** *L.lobata* (♀, gen. slide no. HNUSEL-5320) (scale bars: 0.5 mm).

**Male genitalia** (Fig. 2F). Tegumen is as long as 2/3 of valva, membranous and slender. Valva elongated, narrowed to apex and is slightly swollen at 2/3 to basal part; long setae along ventral margin on the apex to sub-basal part, longer at the median to basal, a star-like, sclerotised with four large spinules on apex and each spinule same in length; costal process as long as valva, broad at 1/3 to apical, apical slightly rectangular with long and straight setae. Vinculum elongated and narrowed to saccus; saccus narrow and short. Aedeagus entirely sclerotised, slightly bent, broad to apex, concave near apex with large spinules, apex highly acute and numerous tiny spinules covered at 1/3 from the apex.

#### Distribution

Korea, Japan.

#### Notes

This species was reported for the first time from Korea by Kim and Byun (2019).

#### Host plants

*Wisteriafloribunda* (Willd.) DC. [Fabaceae] in Korea (in this study). *W.brachybotrys* Siebold & Zucc. [Fabaceae] in Japan (Kuroko 1960, De Prins and De Prins 2011).

### Liocrobyla desmodiella

Kuroko, 1982

1CFD7B91-E343-5902-8F39-75B21648DE00

Liocrobyla desmodiella Kuroko, 1982: 185. TL: Japan. TD: ELKU (Holotype); EIHU, NHMUK (Paratypes).
Liocrobyla paraschista Meyrick: Kuroko, 1960: 2. Misidentification.

#### Materials

- **Type status:** Other material. **Occurrence:** individualCount: 1; sex: male; otherCatalogNumbers: gen.slide no. HNUSEL-5319; **Taxon:** taxonID: HNU_DNA_1860; scientificNameID: *Liocrobyladesmodiella* Kuroko, 1982; phylum: Arthropoda; class: Insecta; order: Lepidoptera; family: Gracillariidae; genus: Liocrobyla ; **Location:** country: South Korea; stateProvince: Gangwon-do; county: Samcheok-si; verbatimLatitude: 37°25’10.63”; verbatimLongitude: 127°54’11.50”; **Event:** year: 2014; month: 7; day: 10
- **Type status:** Other material. **Occurrence:** individualCount: 1; sex: male; otherCatalogNumbers: gen.slide no. HNUSEL-5319; **Taxon:** taxonID: HNU_DNA_1861; scientificNameID: *Liocrobyladesmodiella* Kuroko, 1982; phylum: Arthropoda; class: Insecta; order: Lepidoptera; family: Gracillariidae; genus: Liocrobyla ; **Location:** country: South Korea; stateProvince: Gangwon-do; county: Hoengseong-gun; verbatimLatitude: 37º35'44.60"; verbatimLongitude: 128º14'39.81”; **Event:** year: 2018; month: 8; day: 9

#### Description

##### Redescription

**Adult** (Fig. 1B). Head silvery-white mixed with goldish pale brown; frons white mixed with fuscous and a tuft of blackish-brown near each eye; maxillary palpus white and lateral side fuscous; labial palpus white, apex of first segment fuscous and a fuscous spot on apical of second segment; antenna ochrous and apical of each segment greyish; scape white dorsally with a ventral blackish stripe. Thorax white mixed with pale ochrous and greyish scales; tegula same colouration as thorax and darker grey posteriorly; legs white; fore coxa greyish fuscous with a white band on apical part; fore femur fuscous; fore tibia fuscous, except for ventral median part; fore tarsus with two pale fuscous bands on median and second and third segment to last fuscous; middle femur greyish-ochrous; middle tarsus fuscous at basal part with two fuscous bands on median part and second and third segment to last dark fuscous. Wingspan 7.0-8.0 mm. Forewing ground colour greyish fuscous; a goldish-ochrous stripe along dorsal margin on basal to subapical part; a short white streak on 1/6 and 1/3 to base just above outer marginal stripe; first white costa-stria begins at 1/3 to basal part, stretched along costal margin to median and obliquely reaching to outer marginal ochrous stripe across forewing longitudinally; rather triangular white stria on 2/3 to base; a short and narrow white stria near apex. Hind-wing lanceloate and ground colour grey.

**Male genitalia** (Fig. 2G). Tegumen is membranous, as long as valva or slightly shorter and inner surface is covered with narrow setae sparsely. Valva is somewhat hammer-like, costal margin slightly sclerotised, slightly concave at 1/6 to apex on costal margin and outer margin concave at 1/4 to apex; stout setae along apex to costal margin on inner side of sclerotised area, setae much shorter on basal than apical part, a row of sclerotised teeth on apex to near median part and each tooth minute with slightly blunt apex. Vinculum reduced, rectangular and smoothly narrowed to saccus; saccus wide and apex blunt. Aedeagus slightly sclerotised on median part, bent-shaped, narrowed to apex, apex bevelled and coiled with wrinkles at median part.

#### Distribution

Korea, China, Japan, Russia.

#### Notes

This species was reported for the first time from Korea by Kim and Byun (2019).

##### Enter subsection title

Enter subsection text

##### Enter subsection title

Enter subsection text

### Liocrobyla indigofera

Liu, Wang & Wang, 2018

96F94F4E-F991-56C0-B670-741B85D10CF9

#### Materials

- **Type status:** Other material. **Occurrence:** catalogNumber: HNU_DNA_1858; individualCount: 1; sex: female; otherCatalogNumbers: gen.slide no. HNUSEL-5530; **Taxon:** taxonID: HNU_DNA_1858; scientificNameID: *Liocrobylaindigofera* Liu, Wang & Wang, 2018; phylum: Arthropoda; class: Insecta; order: Lepidoptera; family: Gracillariidae; genus: Liocrobyla; **Location:** country: South Korea; stateProvince: Gyeonggi-do; county: Yeoncheon-gun; verbatimLatitude: 38.076944; verbatimLongitude: 127.136694; **Event:** year: 2018; month: 8; day: 9

#### Description

##### Redescription

**Adult** (Fig. 1C). Head dark grey; frons white with a tuft of blackish fuscous scales below scape; maxillary palpus pale ochrous with black band on apical of each segment; labial palpus white, blackish band on sub-base of each segment with a black spot on apex; each segment of antenna black and much lighter to base; scape fuscous with a dorsal black line. Thorax pale ochrous mixed with blackish tiny scales; tegular dark fuscous; legs white with blackish fuscous rings. Wingspan 6.0 mm. Forewing ground colour fuscous with a bronze dorsal marginal stripe; a white spot on 1/6 to base just above bronze marginal stripe; a short obliquely white stria beginning on bronze marginal stripe at a half of forewing; a white costa-stria at 1/3 to base and reached to 1/5 on width of forewing; more long white costa-stria at a half of forewing and stretched near wing fold; a roundly bent and narrow white stria on apex with a blackish spot on apex between white spot. Hind-wing grey and cilia long.

**Female genitalia** (Fig. 2H). Papillae anales well sclerotised with long setae on apex; apophyses posteriores are more stout and broad than anteriores. Ostium bursae moderated in opening size; antrum well sclerotised as long as apophyses anteriores. Ductus bursae are entirely sclerotised and curved to S-shaped on the median; ductus seminalis originated near the antrum. Corpus bursae membranous, slightly reduced and ovate without signum.

#### Distribution

Korea (new record), China.

#### Notes

This species is reported from Korea for the first time.

#### Host plant

*Indigoferakirilowii* Palib. [Fabaceae] in Korea (in this study). *I.kirilowii* Palib., *I.tinctoria* L. [Fabaceae] in China (Liu et al. 2018, De Prins and De Prins 2011).

### Liocrobyla kumatai

Kuroko, 1982

DA9E8B15-0830-561A-B108-92B8698A9D3F

Liocrobyla kumatai Kuroko, 1982: 185. TL: Japan. TD: ELKU (Holotype); EIHU, NHMUK (Paratypes).

#### Materials

- **Type status:** Other material. **Occurrence:** individualCount: 1; sex: female; otherCatalogNumbers: gen.slide no. HNUSEL-5528; **Taxon:** taxonID: HNU_DNA_1857; scientificNameID: *Liocrobylakumatai* Kuroko, 1982; phylum: Arthropoda; class: Insecta; order: Lepidoptera; family: Gracillariidae; genus: Liocrobyla; **Location:** country: South Korea; stateProvince: Jeollabuk-do; county: Jeonju-si; **Event:** year: 1977; month: 7; day: 13

#### Description

##### Redescription

**Adult** (Fig. 1D). Head white with a dark brown median line; frons white and smooth with a tuft of blackish-brown scales below scape; face white mixed with fuscous; maxillary palpus fuscous and apical ochrous; labial palpus white with a dorsal fuscous band on the first segment at median to apical and a minute ring on the subapical of the second segment; each antenna segment on dorsally white to basal and the rest of them brown and white ventrally; scape white with a lateral fuscous line. Thorax white; tegular pale ochrous and fuscous posteriorly; legs white with fuscous bands; fore coxa milky fuscous with white band on apical part; fore femur fuscous laterally; fore tibia blackish fuscous with two white spots on the basal and median part; fore tarsus with tree fuscous band and the last one most broad; middle femur fuscous laterally; middle tibia with two oblique narrow fuscous bands on 1/3 and 2/3 to base with fuscous apical; middle tarsus with four oblique fuscous bands. Wingspan 6.3–7.8 mm. Forewing ground colour fuscous and bronzy ochrous near apex; white streak on dorsal margin from basal to median with two black spots and most of the outer area tinged with pale ochrous; white costa-stria begin at 1/3 to base, stretched along costal margin over a half of forewing, the width of stria on costal margin almost same as the white streak on dorsal margin, rapidly curved downwards obliquely and reaching to wing fold; second white costa-stria at 2/3 to the base with black stria on inner side; more narrow and slender white stria near the apical part and downwards obliquely near the blackish spot of apex; a slightly elongated horizontally black spot on the apex with two white spots and the lower one near tornus longer than upper one. The hind-wing ground colour is greyish and the cilia are long.

**Female genitalia** (Fig. 2I). Papillae anales elongated caudally, sclerotised, long scales on the apex and stout setae densely on the inner side; apophyses posteriores stout and reduced. Ostium bursae wide at opening; antrum sclerotised and as long as 2/3 of apophyses anteriores. Ductus bursae reduced and membranous; ductus seminalis just below antrum with tiny and minute spinules, sclerotised lines originating from ductus bursae form a ring laterally and the sclerotised ring elongated as long as antrum. Corpus bursae are membranous and ovate.

#### Distribution

Korea, Japan, Russia.

#### Host plants

*Lespedezabicolor* Turcz. [Fabaceae] in Korea (in this study). *L.bicolor* Turcz. [Fabaceae] in Japan (De Prins and De Prins 2011, Ikeda 1995). *Desmodium* sp. [Fabaceae] in Russia (Ermolaev 1987, De Prins and De Prins 2011).

### Liocrobyla lobata

Kuroko, 1960

8467183A-5FAA-5EEA-ADE8-146D5C6CFC6D

Liocrobyla lobata Kuroko, 1960: 5-6. TL: Kyushu, Japan. TD: ELKU (Holotype; Allotype; Paratype).

#### Materials

- **Type status:** Other material. **Occurrence:** individualCount: 1; sex: female; otherCatalogNumbers: gen.slide no. HNUSEL-5320; **Taxon:** taxonID: HNU_DNA_1830; scientificNameID: *Liocrobylalobata* Kuroko, 1960; phylum: Arthropoda; class: Insecta; order: Lepidoptera; family: Gracillariidae; genus: Liocrobyla; **Location:** country: South Korea; stateProvince: Gyeongsangnam-do; county: Chungmu; **Event:** year: 1977; month: 8; day: 14

#### Description

##### Redescription

**Adult** (Fig. 1E). Head white with a tuft of fuscous scales above the scape and a blackish narrow stripe below the scape; frons white and face ochrous; maxillary palpus pale ochrous with a tiny fuscous spot on the apex of each segment; labial palpus white with a narrow apical fuscous ring on each segment; each segment of antenna ochrous inward and greyish outward; scape fuscous dorsally and whitish ventrally with scales sparsely. Thorax white; tegular pale ochrous and fuscous posteriorly; legs whitish ochrous with fuscous bands; fore coxa pale ochrous with lateral fuscous tiny spots and a white band apically; fore femur fuscous; middle femur fuscous and tarsus with oblique fuscous band apically. Wingspan 6.0 mm. Forewing light fuscous with a bronze dorsal marginal stripe; a dorsal marginal stripe occupying near wing fold on basal to 2/3 of the forewing and a half of on apex with some white streaks and blackish spots; first white costa-stria beginning at 1/3 to base, stretched along costal margin near a half of forewing, smoothly downward and reached to bronze stripe obliquely; second white costa-stria narrower than first one; third one slightly bent inwards and apical part acute; an obscure tiny white spot on apex. Hind-wing ground colour is light greyish-ochrous.

**Female genitalia** (Fig. 2J). Papillae anales as long as apophyses anteriores with long setae on apex; apophyses posteriores sclerotised, stout and triangular; apophyses anteriores slender and narrow. Ostium bursae wide at opening; antrum well sclerotised and elongated as long as apophyses anteriores or longer. Ductus bursae 2 times longer than antrum, sclerotised on base to just above corpus bursae and membranous near corpus bursae; ductus seminalis originating just below antrum, base sclerotised, curved and slightly turned back to ductus bursae. Corpus bursae are membranous, ovate and as long as ductus bursae without signum.

#### Distribution

Korea, China, Japan.

#### Host plants

*Puerarialobata* (Willd.) Ohwi, *P.* sp. [Fabaceae] in Korea (in this study; Park et al. (1983), De Prins and De Prins (2011)). P.montanavar.lobata (Willd.) Sanjappa & Pradeep [Fabaceae] in China (Liu et al. 2018, De Prins and De Prins 2011). P.montanavar.lobata (Willd.) Sanjappa & Pradeep [Fabaceae] in Japan (Kuroko 1960, De Prins and De Prins 2011).

## Identification Keys

### Key to the species of the genus *Liocrobyla* in Korea, based on adult

**Table:** 

1	First white costa-stria of forewing elongated downwards over wing fold	2
–	First white costa-stria of forewing short, reduced and does not reach the wing fold	*Liocrobylaindigofera* Liu, Wang & Wang, 2018
2	A median dorsal marginal white stria of forewing stretched upwards near wing fold and narrowed to apex	*Liocrobylabrachybotrys* Kuroko, 1960
–	A median dorsal marginal white stria of forewing stretched outwards parallel to hind margin	4
3	White costal stria of forewing with distinct blackish lines on inner side	*Liocrobylakumatia* Kuroko, 1960
–	White costal stria of forewing without blackish lines or obscure on inner side	5
4	Width of first white costal stria broad occupying 1/4 of forewing	*Liocrobylalobata* Kuroko, 1960
–	Width of first white costal stria narrow occupying 1/5 of forewing	*Liocrobyladesmodiella* Kuroko, 1982

## Discussion

In this study, we report significant findings of leaf miners, specifically within the genus *Liocrobyla* of the family Gracillariidae in Korea. In total, five species of *Liocrobyla*, including a newly-recorded species, *L.indigofera*, have been found in Korea. These findings expand our understanding of the Gracillariidae family's biodiversity in Korea and enhance the global knowledge of this genus, which comprises only nine species worldwide. We provide detailed morphological descriptions, particularly of the adult and genitalia structures, which are important for identification of species within *Liocrobyla.* Additionally, we summarise their distribution and host plants, which contributes to the understanding of their ecology and potential impact on ecosystems. Overall, this research contributes significantly to the fields of insect systematics and ecology, particularly concerning the Gracillariidae family in Korea. It underscores the need for ongoing research to explore and document biodiversity, vital for advancing systematics in the future.

## Supplementary Material

XML Treatment for Liocrobyla brachybotrys

XML Treatment for Liocrobyla desmodiella

XML Treatment for Liocrobyla indigofera

XML Treatment for Liocrobyla kumatai

XML Treatment for Liocrobyla lobata
